# Factors affecting ability of TB patients to follow treatment guidelines – applying a capability approach

**DOI:** 10.1186/s12939-023-01991-7

**Published:** 2023-09-01

**Authors:** B Aravind Chandru, Ravi Prasad Varma

**Affiliations:** Achutha Menon Centre for Health Science Studies, Sree Chitra Tirunal Institute for Medical Sciences and Technology, Trivandrum, Medical College Post Office, Thiruvananthapuram, Kerala India

**Keywords:** Anti-tuberculosis treatment, National TB Elimination Programme, Gender, Socioeconomic deprivation, Equity

## Abstract

**Background:**

Negotiating anti-Tuberculosis treatment is a complicated process comprising daily consumption of multiple medications at stipulated times and dosages, as well as periodic follow-ups and investigations, may not be uniform for all Tuberculosis (TB) patients and some may perform better than others. In this context, we conducted a study in Thiruvananthapuram district, Kerala to ascertain the ability of those suffering from TB to follow treatment guidelines.

**Methods:**

This study used an embedded mixed methods design. We collected cross-sectional data from 135 drug sensitive pulmonary TB patients aged 18 years or above in Thiruvananthapuram, Kerala using a structured questionnaire to get the proportion of patients following all treatment guidelines. We also did eight in-depth interviews (four men and four women) from within the survey sample. The in-depth interviews were inductively analysed for getting deeper insights about reasons for the choices people made regarding the treatment guidelines. Written informed consent was taken from all participants and the study was implemented after the necessary programmatic and ethical clearances.

**Results:**

Of the 105 men and 30 women studied, uninterrupted daily drug consumption was reported by 80 persons (59.3%, 95% Confidence Intervals (CI) 50.8-67.2%). Overall, 38 (28.2%, 95% CI 21.3%-36.3%) persons were able to follow all seven aspects of advised guidelines. Living in an extended/ joint family (Adjusted Odds ratio (AOR) 2.6, 95% CI 1.1-6.0), approximate monthly household expenditure of over rupees 13,500 (AOR 2.9, 95% CI 1.3–6.7) and no perceived delay in seeking initial care (AOR 3.2, 95% CI 1.2–8.7) were significantly associated with following all aspects of treatment guidelines. In-depth interviews revealed reflective treatment related behaviours were influenced by bodily experiences, moral perceptions, social construct of TB, programmatic factors and substance use. Sometimes behaviours were non-reflective also. Programmatic stress was on individual agency for changing behaviour but capability and opportunity for these were influenced social aspects like stigma, gender roles and poverty.

**Conclusion:**

TB patients live amidst a syndemic of biomedical and social problems. These problems influence the capabilities and opportunities of such TB patients to follow treatment guidelines. Interventions should balance focus on individual agency and social abd economic factors.

**Supplementary Information:**

The online version contains supplementary material available at 10.1186/s12939-023-01991-7.

## Background

Anti-Tuberculosis Treatment (ATT) is a difficult process that requires medications, investigations and regular follow-ups for at least six months. Kerala state in India had implemented several initiatives for supporting Tuberculosis (TB) patients with better social inclusion within the decentralised health system framework [1, 2] According to the National TB Prevalence Survey in India 2019–2021, Kerala had the lowest prevalence of microbiologically confirmed pulmonary TB for people 15 years of age and older, at 1510 per million [3]. However, TB control in Kerala has some specific challenges like diabetes, reported among 44% of TB patients in Kerala [4]. The state also reported the highest prevalence of TB among the multidimensionally poor (1590 per 100,000 as compared to a national level of 480 per 100,000, and a state average of 370 per 100,000) in another recent study [5].

The treatment approach changed from intermittent to a daily drug regimen based on patient weight bands in 2017 [6]. Additionally, patients were to be encouraged to have routine follow-up exams and screenings for complications or adverse effects. Some programmatic issues were reported in the initial period after this change [7]. Also, the programme had only been in effect for about two years when the COVID-19 pandemic struck in early 2020. Considering the high prevalence of TB among the multidimensionally poor, the co-occurrence with diabetes, a non-communicable disease of epidemic proportions in Kerala, the recent programmatic shift and the impact of COVID-19 on the health services, we decided to do a study on the ability of TB patients in Thiruvananthapuram, Kerala to follow TB treatment guidelines.

## Methods

### Design

The study used embedded mixed methods along the lines described by Creswell et al., comprising a cross-sectional survey among pulmonary TB patients on symptoms, health seeking, treatment aspects and follow up and in-depth interviews for getting deeper insights on these aspects [8].

### Participant selection

Participants were drug sensitive pulmonary TB patients aged 18 years or above from the NIKSHAY Tuberculosis registry of Thiruvananthapuram who had been on TB treatment under the NTEP for at least one month prior to the interview. The NIKSHAY registry data projected the number of individuals with pulmonary tuberculosis (TB) in Thiruvananthapuram in 2020 as 1,200 [9]. With 70.4% expected to adhere to therapy [10] the sample size was determined to be 135, using OpenEpi application for proportion with finite population correction, with a 95% confidence interval (CI), an absolute precision of 10%, design effect of 1.5, and 20% oversampling for losses. From three randomly selected TUs, 45 patients each were randomly selected, with a replacement strategy of selecting another patient aged 18 years or above from the local health centre level if the patient could not be contacted, did not consent or did not speak the local language.

### Data collection techniques

A structured questionnaire was used to gather the participants’ responses to questions about their symptoms, the process of seeking treatment, the diagnosis, the start of treatment, the follow-up check-up, their experiences with stigma, the support they received from the healthcare system, and their opinions of TB treatment. Eight in-depth interviews were also conducted by selecting four consenting men and four women from the cross-sectional survey. Interview participants were chosen from the cross-sectional survey based on age and sex and interviewed at their homes at a time that was convenient for them. All in-depth interviews were conducted and transcribed into English by BAC. The interview guidelines included questions on diagnosis of tuberculosis infection, the services offered, interactions with the treatment provider or supervisor, efforts to adhere to prescribed medications and follow up, perception of importance of treatment adherence and support for treatment.

### Outcome variables and concepts used

The foundations of this work are grounded in the national programme and concerns on continuity in intake of medications and follow up. The topic was conceptualized through informal interactions with a few frontline programme staff and TB patients. Adherence to drug regimen was one of the topics suggested by programme personnel as useful research. Adherence studies from a biomedical perspective can slip into a “victim blaming” discourse but following treatment guidelines for TB was considered of paramount importance as well. Also, patient narratives suggested the importance of bodily experiences. After considering several theoretical options, Mead’s social behaviourism and the capability approach were considered useful. The capability approach was chosen as it could shift attention from the actual act of drug intake (“functioning”, and risk of “victim blaming”) to capability – choice and opportunity.

The outcome of interest for the quantitative analysis was a composite variable - following all treatment guidelines - (i) procuring drugs as advised; (ii) taking medication daily, not missing medication more than one day in the past one month; (iii) at the right prescribed time (iv) with correct dosing; (v) in one sitting (vi) visiting a testing centre as advised, and (vii) follow-up consultation as advised. This reflected the functioning part of the capability of the patient to follow treatment guidelines. We first considered the COM-B (Capability Opportunity Motivation Behaviour) [11] that allows for automatic processes as well as planned behaviour. Capability for adherence is presented as physical, cognitive and executive; motivation as a reflective or automatic process; and opportunity as physical or social factors influencing adherence behaviour. This model does not consider upstream interactions that are important in research on TB patients and still could slip into victim blaming [12, 13]. Yet, individual factors are important for equity - e.g., impact of household income may not be the same for all individuals within the household. Ecological analysis cannot describe limitations in individual economic autonomy of women and the effect on disability and mortality [12]. Ruger suggests a more balanced model that defines health capability as health agency and health functioning [13]. Biological predisposition, intermediate social context, macro level factors like social, political and economic environment and the health care system interact to influence the individual’s ability to achieve health. It could draw from the individual aspects influencing choices or internalities as well as the relational construct of personhood or externalities that shape behaviours. However, this approach also brings in some trade-off when social and structural factors are reduced for interpretation at the individual level.

### Data analysis

Quantitative data were summarised as means and standard deviations, or proportions. For comparisons, unpaired t test or Chi square or Fisher exact test were used. A receiver operating characteristic curve was used to classify individuals into two expenditure categories to classify individuals following all treatment guidelines. Binary logistic regression was used to compute odds ratios, and multivariable modelling was attempted to predict the outcome using variables that had a p < 0.10 at the bivariable comparison level.

**Fig. 1:**
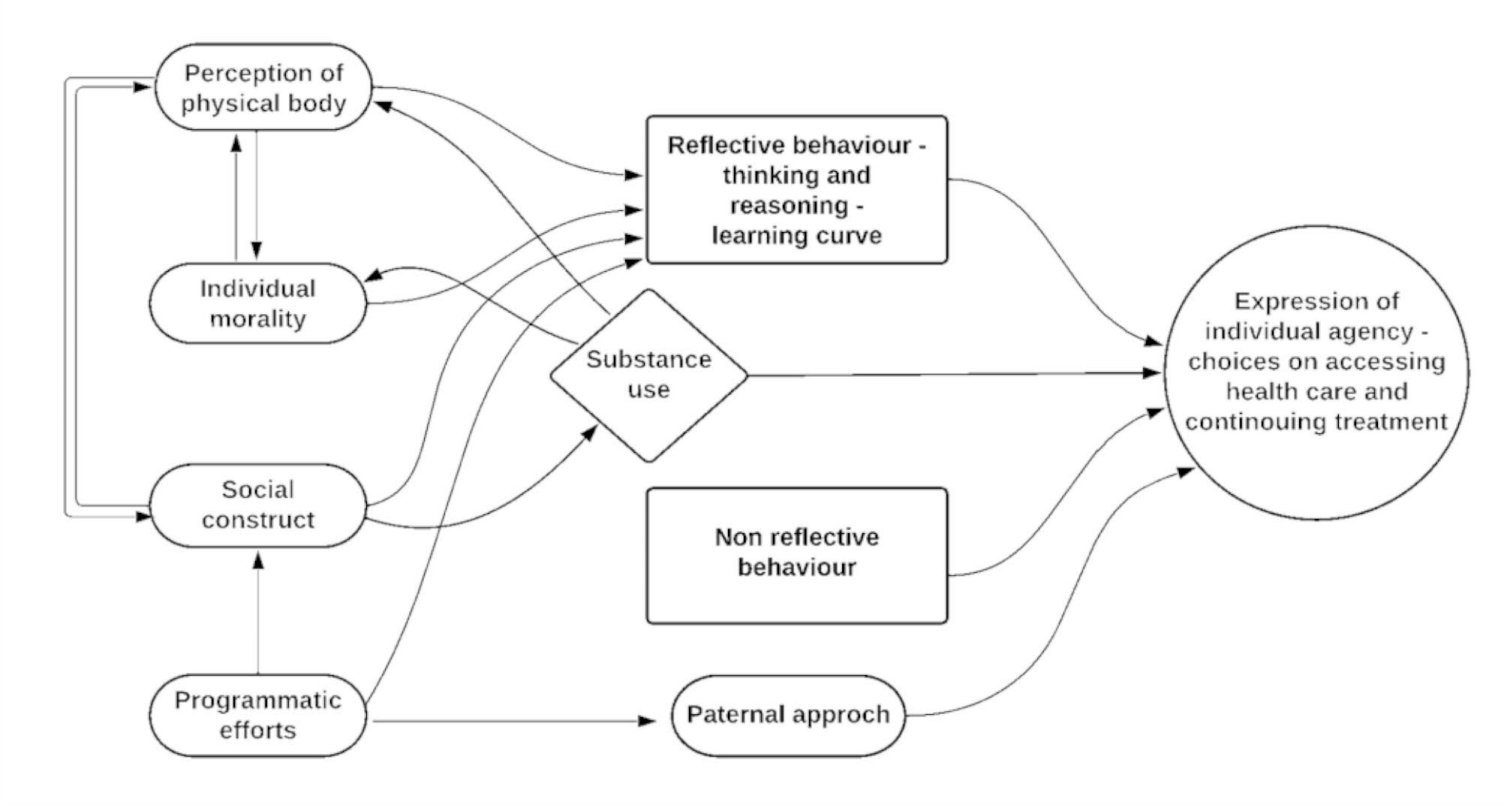
Themes generated from the coding framework depicting factors shaping ability of patients to follow treatment guidelines

The transcripts of the in-depth interviews were translated verbatim into English, then inductive analysis was used to code, categorise and develop themes with which we tried to explain differential experiences of people and differences in the choices they make. BAC performed open line-by-line coding on each transcript. From two transcripts, BAC developed a coding framework. RPV triangulated these findings with the quantitative findings and field notes, evaluated the coding framework for consistency with the codes and transcript portions, and carried out additional code-refining. BAC coded the remaining transcripts adding new codes to the coding framework as needed. Themes to describe perceptions of the disease and treatment experience and how these translated into choices around the prescribed guidelines were developed by BAC using coding framework. (Fig. 1) The process of investigator triangulation continued as each transcript was analysed and both authors debated, named and described the themes and used suitable quotations to illustrate them.

### Reflexivity statement

Both investigators have their basic training in disciplines with predominantly individualised focus (BAC in Ayurveda and RPV in biomedicine) followed by higher education in public health or community medicine with considerable focus on post-positivist approaches. Data collection and analysis of the interviews were driven by our experience from the quantitative data collection process which was structured around the working definition of treatment guidelines we had developed for the study. This may not fully reflect the sequence of thoughts and meanings of TB and its treatment for the participants or findings from constructivist approaches.

### Strategies for trustworthiness

We aimed for credibility through prolonged engagement, persistent observation and investigator triangulation. BAC had prolonged engagement with participants, context and data – starting with field visit, and interaction with programme staff and patients, and later domain experts, and research peers. During the study, each participant was first part of a cross-sectional survey where BAC used a semi-structured interview schedule that lasted 40–45 minutes to complete. In-depth interviews participants were identified from among these persons and the interviews varied from 45 minutes to one hour 45 minutes. Data ranged from spreadsheets for the quantitative survey, audio recordings, photographs, transcripts, and reflections in a field diary. These findings aided data triangulations as well as investigator triangulation where BAC and RPV constantly compared individual interpretations. BAC continues to engage with some of the interview participants for documentation exercises like autobiographies of patient voices.

We consider our findings to be transferable to other settings and other researchers. While patient choices may vary in different settings, it may still be possible to group behaviours as reflective and non-reflective aspects and describe how these will be shaped by the disease and treatment experience. The physical aspect of the disease and treatment experience and the social backdrop surrounding TB will be similar in many settings – poverty, substance use.

Regarding dependability, we expect our findings to be stable. Even if alternate frameworks are used, the essence of the findings will be similar and the variations may be in the interpretations. The authors independently performed reflective assessment of the data and often-expressed viewpoints were combined to arrive at insightful conclusions. Discrepancies were debated and argued and this aided in providing existing data with new context or interpretations. We cite comparable findings from other studies, according to which attention should be paid to structural elements in order to address the matter.

To aid confirmability of our data collection, analysis and reporting, we maintained thorough documentation of all our efforts and kept an audit trail describing our actions at all stages of the study. Peer debriefing was also conducted with faculty members of the host institution, experts in respiratory medicine, and public health experts involved in TB control. All of these experts were considered sufficiently detached from the programme and the setting while giving comments.

### Ethical aspects

The State Operational Research Committee of the National TB Elimination Programme - Kerala approved the study. The Institutional Ethics Committee of Sree Chitra Tirunal Institute for Medical Sciences and Technology, Trivandrum, reviewed and cleared the protocol and tools. (IEC/1816 dated on 02-03-2022). The investigator implemented a written informed consent process prior to each interview and care was taken to ensure that potential for harm or discomfort for participants or the assisting programme staff was kept as minimal as possible.

## Results

The distribution of age, sex, treatment category and diabetes status in the sample and the original sampling frame were comparable. Non-response was analysed in one TU – 45 participants could be selected after approaching 59 (coverage 76.3%). Of the 14 participants replaced, eight were not responding to phone calls by TB health staff for initial consent, two did not give initial consent, one withdrew after initial consent without mentioning any reason, one was traveling, one cited work engagements and one cited health issues. We finally had 105 men and 30 women participating in the study. The Supplementary file shows the frame to sample comparison (Table S1), the socioeconomic characteristics (Table S2), and experiences around TB disease segregated by sex of the participant (Table S3).

Men were more likely to report manual labour as their occupation while women were more likely to be widows. Cough was the commonest symptom. Cough, fever and weight loss were commoner in men but not statistically significant. Loss of appetite was reported by more women than men. Significantly more women approached a private health facility first than men. Faith in providers or institutions mattered significantly more for women. Nearly a third of the participants rated the communication process by the providers and program staff as poor to average (43, 31.9%). Only 10 (7.4%) participants reported being informed about all the following – drug consumption, side effects, follow up testing and consultations, including screening of vision (Table S4). This proportion was significantly higher in women. Side effects were commoner in women (22, 83.3%) than men (68, 64.8%) but not significant. Itching was the commonest side effect (40, 29.6%) followed by vomiting (31, 23.0%), appetite being affected (30, 22.2%) and feeling tired or drowsy (28, 20.7%). Five people (four men and one woman) developed jaundice during treatment. Vomiting was reported by significantly more women than men (Men 19, 18.1%, Women 12, 40.0%, p = 0.012). Overall, more men than women reported that their occupation had been affected (p = 0.073). Stigma was reported by significantly more women than men. This included stigma felt from family members as well as others. Nineteen participants (14.1%) reported having financial difficulties that affected food consumption and treatment seeking (Table S5). Over a quarter of the studied TB patients had multiple comorbidities (37, 27.4%) and this was significantly higher in women. Diabetes mellitus was the commonest comorbidity (Table S6). Alcohol and tobacco use was almost exclusively a male behaviour save for two women who reported smokeless tobacco use.

### Age and sex differences in following treatment guidelines

Table 1 shows the distribution of patients realizing each attribute of the treatment guidelines as well as proportions who realized all seven attributes of the advised guidelines. Daily drug consumption without interruptions was reported by 80 persons (59.3%, 95% CI 50.8–67.2%).

**Table 1:** Proportion of patients realizing the prescribed treatment guidelines

	Men (< 59 years of age) n = 60	Women (< 59 years of age) n = 21	Men (≥ 60 years of age) n = 45	Women (≥ 60 years of age) n = 9
Procuring drugs on time	49 83.1%	17 81.0%	39 86.7%	8 89.9%
Consuming drugs daily	37 61.7%	8 38.1%	29 64.4%	6 66.7%
Consuming drugs a fixed time	46 76.6%	18 85.7%	38 84.4%	7 77.8%
Consuming all drugs in one sitting	51 85.0%	19 90.5%	39 86.7%	7 77.8%
No sub-dosing	49 81.7%	14 70.0%	38 84.4%	5 55.8%
Laboratory testing	44 73.3%	16 76.2%	42 93.3%	7 77.8%
Follow up consultations	46 76.7%	14 66.7%	26 57.8%	5 55.6%
All 7 criteria	20 33.3%	4 19.0%	12 26.7%	2 22.2%

**Fig. 2:**
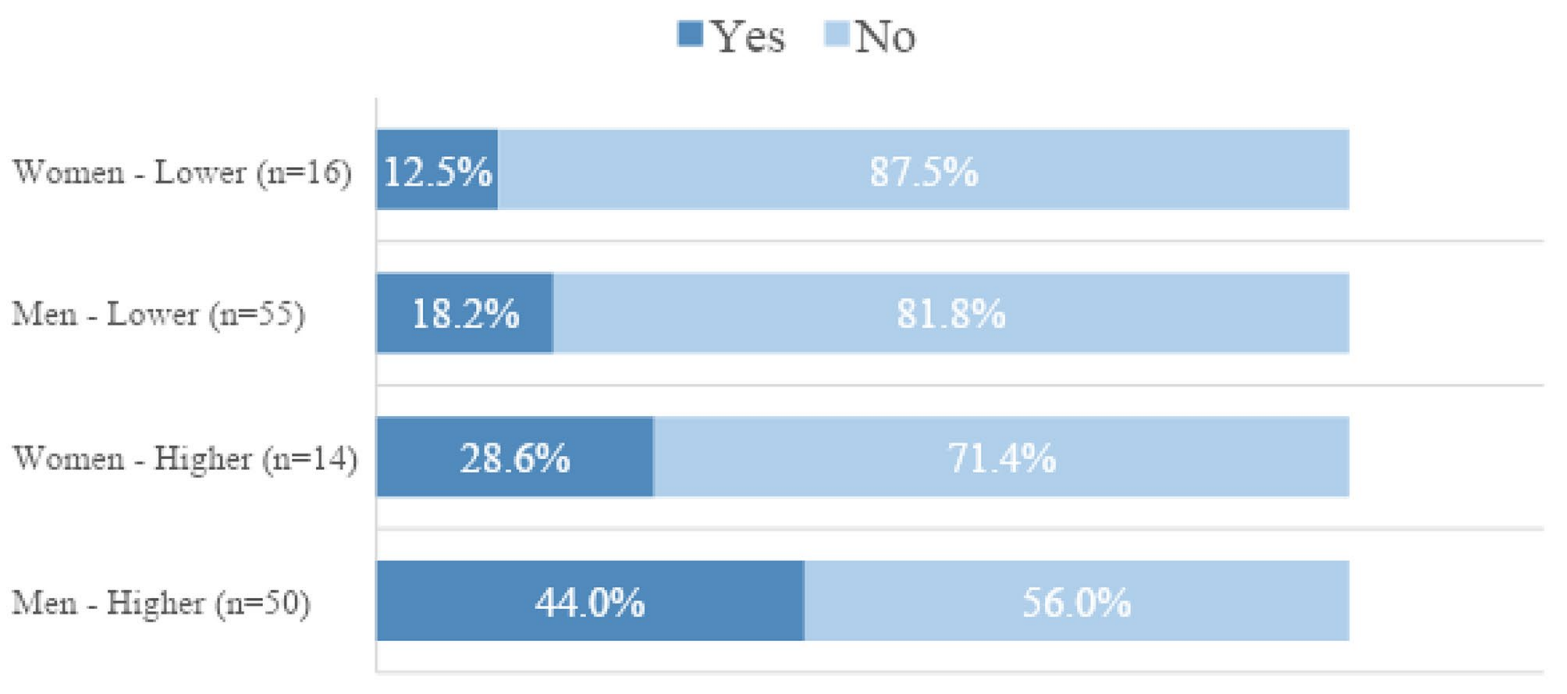
Proportion of men and women of the two studied age groups who could realize all seven criteria of treatment guidelines

Regarding daily drug consumption, younger women had the lowest proportion, about two-thirds of the level achieved by men in the same age group (p = 0.061) Nineteen (65.5%) women never sub-dosed as compared to 87 (82.8%) men (p = 0.042). The difference between men and women was more marked in the older age group (p = 0.072) than the younger age group. Overall, 38 (28.2%, 95% CI 21.3%-36.3%) persons were able to follow all seven aspects of the advised guidelines. Although higher in men (30.5%) than in women (20.0%), the proportions based on sex and age group were not significantly different. Figure 2 shows the proportion of men and women in the two studied age groups who could realize all seven criteria. (Fig. 2) Women in the lower income group had the lowest proportion of persons following all treatment guidelines while men in the higher income group had the highest proportion.

**Fig. 3:**
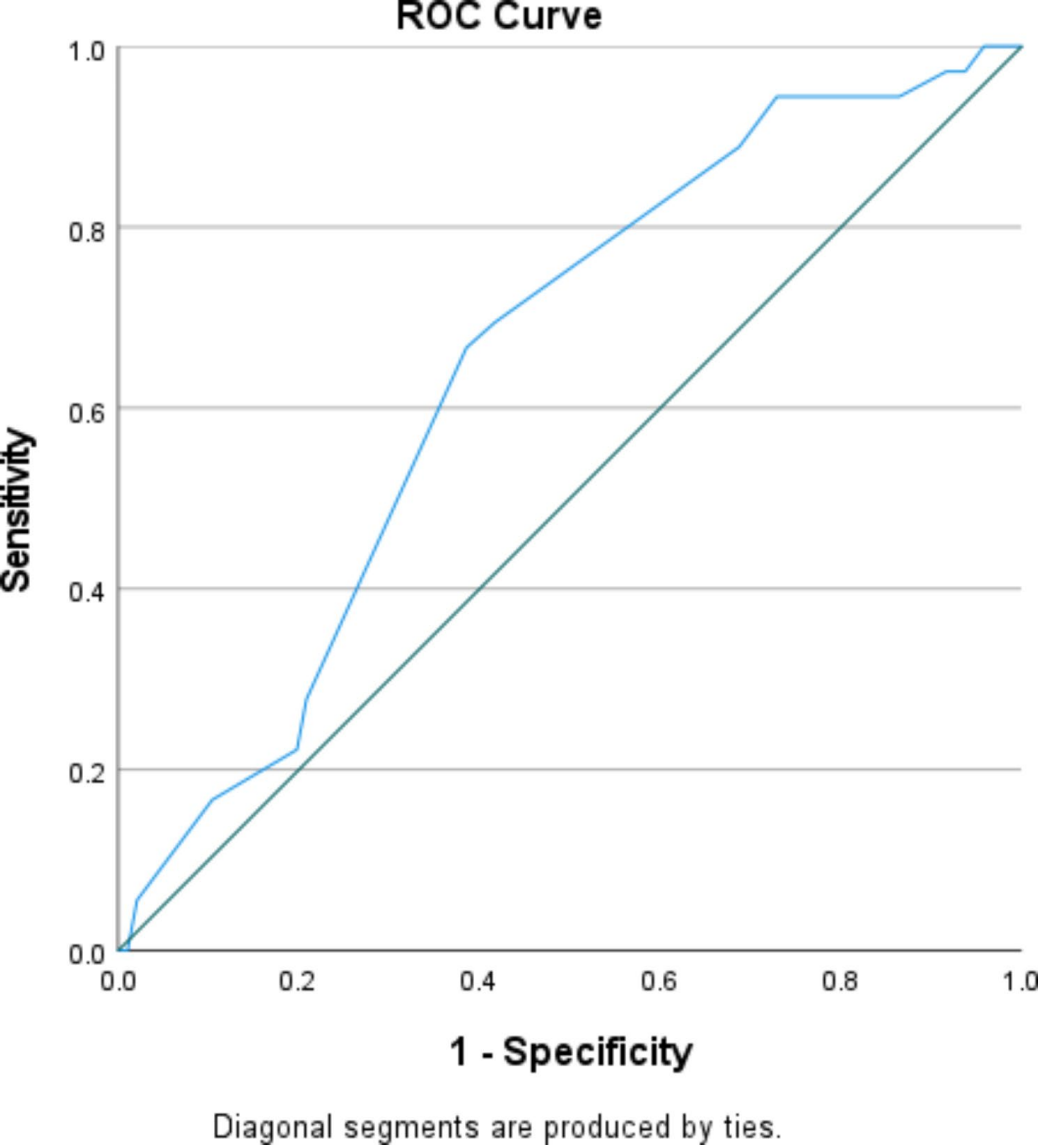
Receiver Operating Characteristic curve of Household expenditure for classifying patients’ practice of following all treatment guidelines

Both sex (13.6%) and age group (3.1%) had inadequate power for further statistical tests for the outcome following all treatment guidelines, but approximate monthly household expenditure (HHE) provided scope for further statistical analysis. Mean monthly HHE was Rupees 15338.9 (SD 6352.9) for participants meeting all seven treatment guideline criteria and Rupees 12458.3 (SD 7499.0) for those who could not (p = 0.039). Considering easier interpretability of categorical variables, an ROC curve (Fig. 3) was used to identify a classification threshold for participants following all treatment guidelines from those who do not based on the monthly HHE. The area under the curve being 0.648 (p = 0.009) the Youden’s J was computed for potential coordinate points and the highest value was 0.281, corresponding to a cut-off of Rupees 13,500. Family expenditure level of over Rupees 13,500 had a sensitivity of 66.7% and specificity of 61.5% for predicting actualization of all requirements of treatment guidelines. For our outcome, this variable had a statistical power of 82.5%.

Table 2 shows the results of logistic regression analysis attempted to compute measures of association for predicting the outcome of this analysis. Being in a household with expenditure over Rupees 13,500 per month meant 3.4 times higher odds (95% CI 1.5–7.5) of following all aspects of the guidelines as being from households with monthly expenditure up to Rupees 13,500. Anticipating the possibility of confounding, crude odds ratios of all other exposure variables were computed, and those with p < 0.10 were included in a multivariable model predicting persons following all treatment criteria. Higher household expenditure categories, belonging to an extended or joint family and a perception of having sought care in time all independently had about three times higher odds of following treatment guidelines.

**Table 2:** Results of logistic regression analysis for prediction of realization of all treatment guidelines by TB patients

Variable	Category	Sample size	Number (%) following all criteria	Crude odds ratio (95% CI)	Adjusted odds ratio (95% CI)
Occupation	Manual labourer	45	8 (17.8)	1	-
	Others	90	30 (33.3)	2.3 (1.0-5.6)	-
Family type	Nuclear/ Lives alone	84	17 (20.2)	1	1
	Extended/ Joint	51	21 (41.2)	2.8 (1.3-6.0)	2.6 (1.1-6.0)
Approximate monthly household expenditure	<= ₹13,500	71	12 (16.9)	1	1
	> ₹13,500	64	26 (40.6)	3.4 (1.5–7.5)	2.9 (1.3–6.7)
Self-perceived timeliness of care seeking	Delayed	111	27 (24.3)	1	1
	Timely	24	11 (45.8)	2.6 (1.1–6.6)	3.2 (1.2–8.7)
Weight band at start of treatment (kilograms)	25–34,35–49	60	12 (20.0)	1	-
	Others	75	26 (34.7)	2.1 (1.0-4.7)	-

Three people were unable to follow any of the seven criteria we studied and their details are given below. The issues mentioned by them included work related problems, stigma, substance use, side effects and difficulty in going to the hospital to collect medicines. (See Supplementary file for details)

### Results of in-depth interviews

We have assigned assumed names to participants to retain some of their individual subjectivities while preserving participant anonymity. Table 3 shows the profiles of the interview participants and their treatment trajectories. We share our representation of one participant in detail before sharing the themes.

**Table 3:** Profiles of participants of in-depth interviews

Assumed name	Participant profile	Institutional visit trajectory (brackets show indication for visit / admission)	Experience in following treatment guidelines
***Women***			
Seema	Woman, homemaker, twenties, relapse, child on preventive treatment	PHI for herself→ SDH for child’s medicines	Irregular timing, treatment completed
Savitri	Widow, Domestic help, sixties, new	Pvt→ DTC→ PHI→ MCH(allergy)→ CDH→ MCH	Following all aspects
Maryam	Widow, sixties, caregiver of cognitively impaired son	PHI→ SDH→ PHI	Sub-dosing
Fatima	Woman, forties, diabetic, hypertensive, dyslipidemic, treatment after default	GH(IP)→ DTC→ PHI→ DTC(itching)→ GH(“Kidney” OP, oedema)→ (Hiatus) → MCH→ DTC→ CDH(IP)	Following all aspects in the second round
***Men***			
Ahmed	Man, forties, diabetic, new, stopped tobacco chewing and alcohol use	Chemist→ SDH→ DTC→ GH(IP)→ PHI	Following all aspects
Manoj	Man, sixties, treatment after defaulting twice; smoker, stopped alcohol,	DTC→ PHI	Following all aspects in the third round
Simon	Man, sixties, new; smoker, alcohol user	4 Pvt→ CDH→ PHI	Sub-dosing, delayed procurement
Sajan	Man, twenties, new miliary TB; quit smoking, alcohol and marijuana	Pvt→ MCH(IP)→ CDH(IP)→ MCH(IP, Cholecystitis)→ (Home)→ CDH(IP)→ MCH→ GH(IP)→ PHI	Following all aspects at the time of interview; later TB meningitis, not other infectious diseases

Abbreviations: MCH – Medical College Hospital; CDH – Chest Diseases Hospital; DH – District Hospital; SDH – Sub-Divisional Hospital; PHI – Peripheral Health Institution; IP – In-Patient; Pvt - Private

### Fatima’s story

Many in Fatima’s family had been affected by TB, including her in-laws, son, husband, brother (died of TB), a neighbour, and brother-in-law. Fatima’s story is also about poor housing and food insecurity, non-communicable disease and substance use. Her home is a rodent infested structure with an uneven mud floor, no cooking gas stove, a toilet with no water connection and a curtain instead of a door, and a strong unpleasant stench. While talking about her experience with TB treatment, Fatima kept expressing idioms of starvation – “*vayar veerkoola*” (the stomach will not distend, meaning not reaching satiety), “*vayar kazhinju pooku*” (loosely translates into the stomach will go on, and meaning continue to live) and “*kalathil venthal*” (if it boils in the pot, meaning if food is available). Food insufficiency meant that taking medicines was difficult.

*They will tell me to eat the tablets, but there is no food to eat after having the tablets*

For Fatima, medicines were not just those for TB. She had been diabetic since her last delivery and had medicines for diabetes mellitus including insulin, hypertension and dyslipidemia.

*I mainly take these three tablets (for TB). Other than this I have seven tablets to take in the morning. It’s written there* (showing the prescription)

Fatima had been informed about the importance of uninterrupted medicine intake for TB.

*That time I was told not to fear, there is only a need to take medicines for six months, there is nothing to be afraid of, to eat food on time, and to control this tablet… Sugar and BP can be managed by one tablet or injection. But this is not like that. If I stop in between, I will have to start the treatment from the beginning.*

She experienced severe itching and oedema during the course of her treatment. In between her complex trajectory of physical complaints, health institution visits and multiple medications, there is a hiatus when she stopped “everything” just after being referred to the “Kidney OP”.

*From that time tablets, medicines, injection everything was stopped (“mudangi”). Completely “mudangi”. TB tablets, Sugar tablets, BP tablets, injection (insulin) everything ...*

Fatima’s neighbourhood has widespread substance use - in-laws were alcohol users; her husband had been arrested for drug trafficking; the interviewer had seen three people drinking near her house before 10 AM; the neighbour who had survived TB had ambled in intoxicated during the interview, requiring the feeble voiced Fatima to shout him down to permit the interview to continue.

### Immediate social context

The immediate social context of the participants was primarily one of poverty and financial insecurity. Fatima’s household was the poorest. Maryam’s household was a shade better with her sons providing her with fish for consumption and sale and a small pension from the Fisheries department. Savitri and her stepdaughter both worked in other households. Sajan had no regular income source but his family supported him for food and medicines. Ahmed worked in a shop for daily wages, and had dependent children whom he could not support. Manoj had a small petty shop, and Francis was a taxi driver and both were independent otherwise and received support. Seema’s husband worked in the Middle East and was supportive and she had the added advantage of living near her maternal home. Families were in general supportive but Fatima and Maryam both mentioned the need for a woman family member during hospitalization.

Maryam: “*I have no one to accompany me to the hospital. Only if we have girl children will they take care of us. If I am not well, only they will have the thought to take Amma to hospital.*”

Maryam also used to forgo care if her granddaughter was not around to take her to the hospital.

Maryam: “*I told her that, if I go alone, I can’t understand anything that they say*”.

People were extremely conscious about keeping their diagnosis private except to a select few persons.

Manoj: “*I keep the tablets inside this (a blue pouch) ... so that I don’t have to show it to anyone… No one in my neighbourhood knows about this*”

Precariousness of work was one of the perceived threats of loss of confidentiality of TB status. Ahmed’s shop owner does not know the diagnosis but a co-worker does. The owner thinks his difficulties were due to the “sugar” problem.

Simon: “*Till now no one knows… Because this disease is spreading in nature, friends and others will fear to mingle with me. I have a taxi and autorickshaw, so people will not call me if they know I have such a disease. That’s why I haven’t told anyone.*”.

For all men, substance use was enabled by their peers. After diagnosis, participants mentioned avoiding peers because of the TB or to stay away from substance use.

Sajan: “*I used to smoke cigarettes and weed. I stopped both. I used to smoke till two days before I got admitted. When I was not well, I used to call my friends and they use to bring me cigarettes…*”; after treatment initiation, “*…When my friends called me, I told them I was out of town.*”

### Themes

TB patients may or may not follow the advice given as per the TB treatment guidelines. At times this represents reflective behaviour arrived at based on one’s knowledge formed through own experience or interactions with others. These experiences shaping this knowledge include the (i) perceptions of one’s physical body, (ii) individual morality where the moral gauge may be indicated by emotions, (iii) social construct of TB, and (iv) programmatic efforts. Substance use experience was interconnected to the themes on the body, morality and social construct but not limited by any of these and therefore we mention it as a separate theme. Non-reflective behaviours were also identified and labelled as such.

### Perceptions of the physical body

For a person with TB, the perceived physical body is the space where the discomforts start manifesting, and affects routine functioning. The body undergoes tests, and is managed through food intake, medicines and follow up tests. Symptoms of ill health were often first realized when they interfered with normal routines, alongside validation by peers of their sense of something being wrong. Participants shared many distressing bodily experiences particularly Sajan who had miliary TB and Fatima who had multiple problems. Difficult experience of medication intake ranged from swallowing the “big tablet” to the bodily effects that followed and the “power of the tablets”. For men this power interfered with alcohol intake. For women, it made them exhausted, causing difficulties in carrying out routine work. For Maryam, the tablets were “hot”. With improvement in diet, medication intake became easier, and recovery was indicated by symptom relief, restoration of the routine or activity, weight gain, and validation by others. Both Ahmed and Sajan wanted to return to “normal” functioning. Seema, a re-treatment patient, just wanted the pills to be over.

Ahmed: “*Only thing I knew from two three people was that my body was getting lean and weak.*”; on the *power of medicines* – “*everything around feels revolving…After being able to eat properly there were no such problems*”

Perceived side effects that affected functioning may lead to stoppage of medications as was the case with Manoj who had defaulted treatment twice in the past.

Manoj: “*Because if I consume the tablets, I am not able to walk. Breathing difficulty…Not able to lift any weight, I couldn’t even carry a bucket of water.*”

### Individual morality

A diagnosis of TB often evoked strong emotions and considerable stress, recurringly connected to a reflection upon oneself. People felt sad, depressed, guilty, angry, or lonely but one person was stoic about it. The guilt could stem from one’s own principles like religious belief, or from what relatives or others (including health care providers) said pertaining to their substance use or past behaviour with respect to medication intake or other a bad horoscope.

Ahmed: “*…only when we get it will we know it is due to all those bad things which we did in the past…Because I went in a bad way (alcohol use) I got this and food habits are also a part of that.*”

Moral reasons cited for taking good food and treatment, stopping substance use and other adjustments (mask, restricted contact) included needing to return to normal, or to be there for their family. Having to be there for someone was mentioned by all four women interviewed, and by one man as well.

Fatima: “*I wish to live... They won’t survive without me. I should live to sustain the other three in this house*”

Maryam: “...*if I die, who will take care of him?*” (son with intellectual disability).

Also, people did not want to transmit the disease to others, their loved ones or anyone else.

Simon: “Also, I pray to God that no one should get disease from me. I won’t even kiss my *muthu* (translates into pearl, he meant his granddaughter), because it spreads”

### Social construct of TB

This theme refers to descriptions of TB that emerge from interactions between individual and groups. Unhealthy diet or alcohol use led to TB but smoking was not mentioned as a cause. Symptoms of TB include becoming lean, weak, and having cough with blood in sputum. Historical knowledge of TB testing that emerged was *Pulayanarkotta*, the Government Chest Diseases hospital which was an erstwhile sanatorium. TB can be spread by germs from the patient to others. Proper food (milk, egg white, meat, and fruits) and medicines can make people better. Children are to be protected from infection, an aspect that often led to self-stigma or discrimination.

Ahmed: “*My friend’s friend once had TB, he took medicines for 7–8 months and was cured, like that they said I will also get cured…When I told her (his wife), she said I got TB due to drinking.*”

### Programmatic efforts

The programmatic efforts were often paternal but health providers were benevolent in general. They diagnose TB, prescribe and provide tablets, inform on financial aid, admit if needed (sometimes even for the food as in Fatima’s case), teach, and scold when participants go (or were expected to go) off track. Phrases like “thick sputum from deep inside the chest”, “control (uninterrupted intake) the medication”, “not to smoke or drink” and “six months” were repeated often. Respectful providers were appreciated. For patients, a relationship of mutual respect with treatment providers helped them cope with the uncertainties and evolving physical experiences arising during the long duration of treatment and the changes in their disease experience. However, the ultimate process of taking medicines, eating good food, overcoming substance use issues and seeking care for health problems rested on the individual agency of the participant. Also, barriers may be present when household members like children are advised to take preventive therapy. Quotes pertaining to this theme are listed in the Supplementary file.

### Addictions

All four men interviewed had substance use behaviours and quitting, if at all, was either during the pre-diagnosis suffering phase or at the start of treatment. Sajan quit alcohol, tobacco and marijuana after the hospitalization phase at the start of treatment on his own although he was offered deaddiction services. Substance use, particularly alcohol was considered a cause for the disease or a cause for interaction with the medicines.

Simon: “*I took the tablets and after one hour I went to drink with friends. At night I vomited blood…The power of tablets will cause reaction with alcohol. So, I won’t mix both…I rest my body* (stop tablets) *one day prior to having drinks… so that the next day I can drink*”

Manoj: “*Few minutes after drinking I started to sweat, there was some “vepralam”* (expression of discomfort). *I was feeling “orumaatiri”* (expression of discomfort). *I was out... The tablets and alcohol won’t go together. Therefore, I stopped drinking.*”

Fatima mentioned the alcohol use of her in-laws when she mentioned their TB. She also indicated her husband’s alcohol use as well as his police arrest for drug trafficking when talking about his TB. Savitri also mentioned her late husband’s alcohol use as the reason for his TB recurrence.

## Discussion

This mixed methods study on the ability of TB patients to follow aspects of treatment guidelines under the NTEP in Thiruvananthapuram district found several factors that influence people’s choices related to their practices. Clinical improvement by treatment motivated patients to continue treatment, as they experienced a perceptible benefit. Free diagnosis and treatment is a public health good as it saves lives, and prevents the spread of disease to others from infected persons. Patient agency and motivation help in meeting treatment needs, but the physical experience of TB, diagnosis and treatment can be complicated due to severity, co-morbidities like diabetes, and side effects of drugs. Psychologically, TB patients may be under stress, have many negative emotions including guilt and possibly depression. Immediate social structures like family, peers, workplace, neighbourhood and religious groups may offer experiences ranging from very supportive to discriminating. For men substance use is a phenomenon deeply intertwined with all these aspects – physical, psychological and social. Our findings on perceptions and practices on alcohol use and TB medication are similar to Nichter’s work from the Philippines [14]. The effect of gendered roles and expectations as well as the difference in symptoms we found are similar to other studies on gender differences of TB [15].

Health providers are generally paternalistic, but encounters with TB programme staff are somewhat different from the usual anonymity people face when visiting health institutions. Participants described, being seen, acknowledged, listened to, responded to when having doubts, and respected in terms of privacy and confidentiality, illustrating “treatment support” for TB. Respect, and good communication facilitated trust. There was a learning curve for participants during treatment, of a few weeks at least, during which support for various issues was needed. During this phase, to be recognised, listened to, and responded with solutions that worked for them was as important as being examined, told, and taught.

There were negative encounters too where patients were discriminated against. Even within positive encounters, health providers seemed to buttress health messages on patients’ guilt – using guilt to influence agency seemed to be an easy option. There were some health system related barriers if multiple provider consultations in different institutions had to be done or if hospital admission was required, similar to those reported by Karim et al. [15]. Health care workers’ reassurance of treatability of TB is well documented in literature [16]. Discussion happens with the patients on treatment support and patient choices are generally respected and that is in line with recent research recommendations [17].

The role of social factors on patients’ capabilities and opportunities for making treatment related choices was evident. As seen from Fatima’s story, people from poor households have greater contact with TB and consequently higher risk of the disease. The discrediting experiences TB patients face reduce them to “tainted” persons [18]. Thus, once the disease sets in, it affects their social capital through inability to work due to social or physical reasons. Home based women may also may face disadvantages in the social relationships on which they are dependent for their means. Factors like financial hardships due to job insecurity, cost of travel and medical costs, stigma, increased isolation from family members were mentioned by Addo et al. in their qualitative study on patients’ experiences living with TB and its treatment [16]. Fear of unintentional disclosure was reported from that study as well, as was the advantage that COVID-19 restrictions like masks gave for TB patients [16].

Addo et al. attributed low adherence to substance use, low levels of education and family support, lack of sound information sources like smartphones, and limited understanding of drug resistance as added factors [16]. This approach suggests that interventions at the patient capability level can work for adherence [11]. This conventional COM-B approach focuses on paternalism rather than balancing with autonomy considerations. Individual bodies respond directly to physical, biological stimuli, and social position occupied by a person and group patterning of individual pathologies reflect inequalities.

Given our findings of complexity of the medical and social situation TB patients live in, we feel that this strand of thought about information provision through smartphones is problematic and somewhat like the immodest claims of causality that Farmer warned about [19]. Farmer reminds us of Rene Dubos’ work on the social nature of TB and points out that there are larger forces that make erratic drug consumption more likely in some communities than others. Farmer also warns against exaggerating the agency of TB patients to comply, while disregarding societal and health system failures and focusing on non-adherence, claiming that adherence was driven by economic factors more than cognitive ones. Our findings also suggest that focusing on cognitive interventions for behavioural change may not be sufficient for TB control given the “very real constraints on agency” (Farmer) that TB patients have as many of the factors are beyond their control.

Yet, patients do make choices for themselves. Kelly et al. describes the life world of an individual as a space where one notices, understands, makes interpretations of the world, judgments about themselves and others and makes decisions based on this [20]. This model focuses on individual agency and individual behaviours, challenging the assumption that societal structures determine or programme human behaviour. But it is a four-vector model of public health that takes into account structural and social factors, including population, environmental, organisational and social vectors. Intervention is focused on behavioural change. Ruger’s capability model [13] is more balanced in this regard as it does not compromise attention on individual agency and difficulties of some people in adhering to treatment regimens. This model recommends studying the interaction of social and structural factors considered irreducible to the individual level to cause differences in individual capabilities, thus not assuming the effect of a collective attribute to be uniform to all individuals in the collective [13] similar to the argument raised by Ballantyne [12].

Our choice is not necessarily a vindication of one theoretical or methodological approach – there are alternative methodologies and alternative interpretations of our own text and data. The lack of such considerations is also not a discounting of the existence or importance of such methodologies and interpretations. The health equity framework of Peterson et al. could be a useful way to look at the findings again as it covers individual factors, psychological pathways, relationships and networks, systems of power [21]. However, the capability approach provided an easier knowledge space for us to incorporate the centrality of perceptions of the physical body in our report. Another useful lens to relook at this topic would be an intersectionality informed approach. A responsible use of this approach would require a better recognition of systems of power, privilege and oppression and how they shape psychological phenomena. An intersectionality lens would also possibly warrant a larger sample size for a mixed methods study and this was beyond the scope of the present attempt.

Applied health research with origins in medical anthropology increasingly points to upstream structural and socio-political determinants as more important than the downstream personal choices and biological factors for conditions like TB that have harmful interactions with other diseases and social conditions. This situation, called syndemic vulnerability, has been raised as a human rights issue [22]. Research into TB and comorbidities has increased extensively due to HIV infection or Diabetes Mellitus. But research attention needs to extend to the social aspects of these synergistic epidemics often called syndemics [23]. According to Mendenhall et al., a syndemic is a collection of epidemics brought on by unfavourable social and structural factors and these constitutive epidemics interact to cause excessive morbidity and death [24]. Hossain et al. mentions syndemics as multimorbidity with intertwined biological, psychological, social, structural and other contextual factors that are in itself laced with inequity [25, 26]. Syndemics are often characterised by systematic exclusion, policies that drive disproportionate wealth distribution and lack of access to resources, but have not gotten the attention they deserve.

Our findings reinforce the need for continuous ongoing patient support for helping patients in adhering to their treatment plans. There is a need to explore interventions that may immediately allay problems of the most vulnerable patients - for example through community kitchens to address food insufficiency. The programme will be truly patient-centred only if the real-world issues of the patients are recognised and addressed. Also, further research is needed into TB relevant policies like urban housing, livelihoods and food security.

### Limitations

We did not explore practices around other medications (e.g., for diabetes) and that might have been relevant to the TB treatment experience as well. We also did not capture provider perspectives or explore macro-level social, political and economic aspects beyond the immediate context, and that would limit our analysis. Despite these limitations, we feel that we were able to comprehend many of the facilitators and barriers TB patients are likely to face when expected to exert their agency for following all guidelines related to treatment and follow up.

## Conclusion

The current forms of screening, diagnosis, treatment and support for TB, despite limitations, are helping individuals recover and restore their lives to pre-disease states. Free diagnosis, treatment and good support for TB patients is a public good where many people beyond the immediate beneficiaries get advantage. Educating TB patients to follow all aspects of the treatment guidelines – medication management (procurement, intake, care for side effects), investigations and follow up – is very important, but is heavily dependent on the individual agency of patients. Expressing individual agency is challenged by complex factors often beyond individual control. The distribution of capabilities and opportunities for TB patients to exercise their individual agency into functioning in terms of completing treatment as prescribed is not uniform. Education and motivation of TB patients for treatment adherence might not be enough for an epidemiological shift to happen. Also, TB is often part of a syndemic of poverty, substance use, non-communicable diseases like diabetes mellitus and quite possibly depression, with variations stemming from socioeconomic disadvantages, gendered roles and stigma being unfair and unjust. Given the threat of multidrug resistant tuberculosis emergence in such disadvantaged communities, failure to realise the social and structural factors that drive the syndemic in which TB coexists and influence patient choices regarding following treatment guidelines may make TB elimination an implausible dream.

### Electronic supplementary material

Below is the link to the electronic supplementary material.

Supplementary Material 1

## Data Availability

The corresponding author will provide the transcripts, data set, and analysis of this current work on reasonable request.

## List of abbreviations

AOR: Adjusted Odds Ratio
ATT: Anti Tuberculosis Treatment
CI: Confidence Intervals
COM-B: Capability, Opportunity, Motivation, Behaviour
HHE: Household Expenditure
NTEP: National Tuberculosis Elimination Programme
ROC: Receiver Operating Characteristic
TB: Tuberculosis
TU: Tuberculosis Unit
