# Supplementary material for: Factors affecting ability of TB patients to follow treatment guidelines – applying a capability approach
Source: Int J Equity Health. 2023 Sep 1;22:176. doi: 10.1186/s12939-023-01991-7 (PMC10474720; doi:10.1186/s12939-023-01991-7)
Supplement: Supplementary file 1 — Supplementary Material 1 [file 12939_2023_1991_MOESM1_ESM.docx]

**Factors affecting ability of TB patients to follow treatment guidelines – applying a capability approach – Supplementary file**

**Supplementary tables**

Table S1. Characteristics of sampling frame and study sample

| **Variable** | **Categories** | **Frame**  **N=535** | **Sample**  **n=135** |
| --- | --- | --- | --- |
| Age and sex categories | Men up to 60 years | 244  45.6% | 60  44.4% |
|  | Women up to 60 years | 78  14.6% | 21  15.6% |
|  | Men 60 years of age and older | 172  32.1% | 45  33.3% |
|  | Women 60 years of age and older | 41  7.7% | 9  6.7% |
| Treatment categories | New | 476  88.8% | 119  88.1% |
|  | Retreatment | 47  8.8% | 16  11.9% |
|  | Drug Resistant TB treatment | 12  2.4% | - |
| Diabetic status | Diabetic | 243  45.4% | 68  50.4% |
|  | Nondiabetic/ No information | 293  54.6% | 67  49.6% |

Table S2. Sociodemographic characteristics of the study participants

|  | **Men**  **n=105** | **Women**  **n=30** | **p value** |
| --- | --- | --- | --- |
| Rural | 42  40.0% | 13  43.3% | 0.743 |
| 60 years or above | 45  42.9% | 9  30.0% | 0.205 |
| No formal schooling | 12  11.4% | 6  20.0% | 0.232 |
| Manual labourer | 39  37.1% | 6  20.0% | 0.079 |
| Approximate monthly household expenditure (Mean, SD) | 13063 (7415) | 13948 (6952) | 0.566 |
| Extended / Joint family | 37 3  5.2% | 14  46.7% | 0.255 |
| Currently married and living with partner | 82  78.1% | 17  56.7% | 0.019 |

Table S3. Symptoms at presentation and treatment characteristics

|  | **Men**  **n=105** | **Women**  **n=30** | **p value** |
| --- | --- | --- | --- |
| Retreatment | 13  12.4% | 3  10.0% | 0.999 |
| In continuation phase | 76  72.4% | 22  73.3% | 0.918 |
| Weight band (25-34 and 35-49 kilograms) | 44  41.9% | 16  53.3% | 0.267 |
| Cough | 99  94.3% | 25  83.3% | 0.066 |
| Fever | 52  49.5% | 10  33.3% | 0.117 |
| Weight loss | 69  65.7% | 15  56.7% | 0.363 |
| Haemoptysis | 26  24.8% | 7  23.3% | 0.872 |
| Loss of appetite | 21  20.0% | 11  36.7% | 0.058 |
| Breathlessness | 29  27.6% | 9  30.0% | 0.798 |

Table S4. Health care seeking and communication at the time of TB diagnosis

|  | **Men**  **n=105** | **Women**  **n=30** | **p value** |
| --- | --- | --- | --- |
| Private health facility accessed first | 40 38.1% | 18  60.0% | 0.033 |
| Faith in provider or institution as a reason for first choice | 26  24.8% | 14  46.7% | 0.020 |
| Perceived delay in health care seeking | 89  84.8% | 22  73.3% | 0.149 |
| Rating of communication process at TB diagnosis (poor to average) | 20  28.6% | 13  43.3% | 0.126 |
| Communication complete | 5  4.8% | 5  16.7% | 0.043 |

Table S5. Social experiences during the course of treatment

|  | **Men**  **n=105** | **Women**  **n=30** | **p value** |
| --- | --- | --- | --- |
| Stigma | 23  21.9% | 13  43.3% | 0.019 |
| Change in occupation | 68  64.8% | 14  46.7% | 0.073 |
| Financial difficulties affecting food/ treatment | 15  14.3% | 4  13.3% | 0.999 |

Table S6. Comorbidities among the studied TB patients

|  | **Men**  **n=105** | **Women**  **n=30** | **p value** |
| --- | --- | --- | --- |
| Diabetes mellitus | 52  49.5 | 15  50.0 | 0.963 |
| Hypertension | 15  14.3 | 10  33.3 | 0.018 |
| COPD | 13  12.4 | 9  30.0 | 0.046 |
| CAD | 10  9.5 | 2  6.7 | 0.999 |
| Multiple comorbidities | 24  22.9 | 13  43.3 | 0.027 |

**Details of three participants who were not able to follow any of the seven attributes of treatment guidelines**

1) 54-year-old man with no formal schooling, married, self-employed, mentioned that his work is affected, regularly consumes alcohol and smokes, and refuses to go to hospital due to fear of being recognised and stigma.

2) 47-year-old woman – educated up to primary school, a manual labourer, currently married, unable to tolerate the tablets - feeling tired, and stomach pain; she stopped when she travelled away from home, and also when she had fever. She feels stigmatized and mentioned a distressing situation where “the hospital attendant asked others to move to a side and told them I have TB.”

3) 75-year-old widow in a rural area, has no formal schooling, living in a household with just herself and another relative she has to depend on to collect the medicines as she has difficulty in traveling to the hospital. She had vomiting, and developed jaundice, causing interruption to treatment.

**Quotes demonstrating findings under the theme programmatic aspects**

**Scolding and warnings**

Sajan: “*He scolded me for tattooing. I told him that I am a tattoo artist and work in a tattoo shop. And I did all these tattoos on my body while I was studying tattoos.*”

Simon: “*They (CDH) hadn’t told me that I should eat three tablets a day, or maybe I forgot, I took one tablet a day for 28 days. The doctor Amma in this health center (PHI) told me to take three tablets together at a time…From here they told me, doctor Amma scolded me for that and the sisters were laughing at me.*”

Sajan: “*he said if I don’t give mucus for testing, they will collect by inserting a tube*”

Manoj: “*I got a phone call from the hospital. They told me there will be many problems if I am not taking the medicines. This disease spreads, so if I am not taking medicines then they will inform station (police station). And told not to create such a situation.*”

Savitri: “*She talked in anger, because my daughter forgot to test her blood.* (Contact screening)”

**Communication gap**

Manoj: “*If I had told my difficulties earlier in the first treatment itself and continued medicine properly then I would have been free before itself.*”

Stress on individual agency

Ahmed: “*They (programme staff) give a very good class not to go back to addictions.*”

**Acknowledged and helped**

Ahmed: “*Asked me to call him and visit him if there are any difficulties. And told he will provide maximum help… He helps me continually for everything.*”

Sajan: “*That doctor (*the one treating TB*) also showed anger towards me, as I had so many tattoos...When I told am a tattoo artist, he was okay. After that he talked in a very friendly way. Whenever I go, he recognises me.*”

Manoj: (On TB Health visitor) “*He talks to me like a brother. Today I am talking to you like this just because of him*”

Seema: “*I always got good care. She replies to every doubt we ask.*”

**Respect for privacy and confidentiality**

Ahmed: “*They have told me there is no need to tell others about this disease and they won’t tell anyone about this.*”

Seema: “*From hospital JHI sir asked me whether they need to inform ASHA workers in my area. I asked him not to tell and they respected that. I am satisfied* (with the support received)”
